# An unusual cause of inspiratory stridor in the newborn: congenital pharyngeal teratoma – a case report

**DOI:** 10.1186/s12887-015-0539-9

**Published:** 2016-01-05

**Authors:** Anna Posod, Elke Griesmaier, Andrea Brunner, Claus Pototschnig, Rudolf Trawöger, Ursula Kiechl-Kohlendorfer

**Affiliations:** Department of Pediatrics II, Neonatology, Medical University of Innsbruck, Innsbruck, Austria; Department of Pathology, Medical University of Innsbruck, Innsbruck, Austria; Department of Otorhinolaryngology, Medical University of Innsbruck, Innsbruck, Austria

**Keywords:** Inspiratory stridor, Newborn, Teratoma, Pharyngeal, Airway obstruction

## Abstract

**Background:**

Neonatal inspiratory stridor is an important examination finding that requires immediate and adequate evaluation of the underlying etiology. Depending on the severity of the airway obstruction and the presence or absence of associated symptoms such as respiratory distress and feeding problems, early initiation of a complete diagnostic workup can be crucial. The most common cause of neonatal inspiratory stridor is laryngomalacia, however, several differential diagnoses need to be investigated. More rare causes include oral or laryngeal masses. Teratomas of the head and neck region are one of the most unusual causes of respiratory distress during the neonatal period. We present a case of a mature teratoma in the oropharynx presenting with airway obstruction in a newborn infant.

**Case presentation:**

A four-day-old female Caucasian infant was admitted to the neonatal intensive care unit of our hospital because of inspiratory stridor and profound desaturations while feeding. Diagnostic workup by ultrasound, magnetic resonance imaging and flexible endoscopy revealed a pediculated lesion in the pharyngeal region causing intermittent complete airway obstruction. The mass was surgically removed by transoral laser resection on the seventh day of life. Histological evaluation was consistent with a mature teratoma without any signs of malignancy. The further hospital course was uneventful, routine follow-up examinations at 3, 6 and 9 months of age showed no evidence of tumor recurrence.

**Conclusion:**

Neonatal stridor is a frequent symptom in the neonatal period and is mostly caused by non-life-threatening pathologies. On rare occasions, however, the underlying conditions are more critical. A careful stepwise diagnostic investigation to rule out these conditions, to identify rare causes and to initiate early treatment is therefore warranted.

## Background

Inspiratory stridor is defined as an abnormal, high pitched respiratory sound resulting from turbulent air flow during inspiration when a partial obstruction of the supra-glottic or glottic airway is present [1]. Neonatal inspiratory stridor is an important examination finding, implying airway obstruction that requires immediate and adequate evaluation of the underlying etiology [2]. Upon admission of a newborn infant with inspiratory stridor, several differential diagnoses must be considered.

In the newborn period, laryngomalacia is the most common cause of inspiratory stridor, which worsens with agitation, after feeding, and in supine positioning [3–5]. Other causes (such as cardiovascular anomalies, vocal cord paralysis, et cetera) are far less common, but pose the risk of complete airway obstruction [3, 4, 6]. Thus, a thorough examination and early initiation of a complete differential diagnostic workup are required.

Among the more unusual causes of airway obstruction in the neonatal period are masses of the head and neck region. Neonatal tumors are rare and estimated to occur approximately once in every 12500–27500 livebirths [7]. Teratomas are one of the main tumor types encountered in the newborn period [8]. They are neoplasms deriving from more than one primitive embryonic layer (ectoderm, mesoderm, endoderm), represent approximately one third of all neonatal tumors and typically arise in the sacrococcygeal region or the gonads [8–10]. The head and neck region is seldom involved; oropharyngeal and nasopharyngeal teratomas account for less than 10 % of all neonatal germ cell tumors [11, 12]. In the following, we present the rare case of an oropharyngeal teratoma causing inspiratory stridor and critical airway obstruction in a newborn.

## Case presentation

A 4-day-old female Caucasian infant was admitted to the neonatal intensive care unit of our hospital because of inspiratory stridor and desaturations while feeding. The girl had been delivered spontaneously at another hospital at 40 weeks 5 days’ gestation to a 32-year-old primigravid mother after an uneventful pregnancy with normal routine prenatal ultrasounds. Rupture of membranes occurred one hour prior to delivery. The infant weighed 3300 g (31st percentile) at birth, and the Apgar scores were nine at 1 min and 10 at both 5 and 10 min. Umbilical cord arterial pH was 7.32; umbilical cord base excess was −3.0 mmol/l.

After birth, the infant was admitted to the newborn nursery, where she was given the first dose of oral vitamin K and received routine care. The first 3 days of life were uneventful. On the fourth day of life, the patient presented with cyanosis while feeding. Upon examination, an inspiratory stridor was noted. Visualization of the oropharynx was attempted, but during examination an episode of deep cyanosis requiring mask/bag ventilation for approximately 30 s occurred. After stabilization, the patient was transferred to our hospital by helicopter and admitted to the neonatal intensive care unit.

On admission, the infant’s temperature was 36.7 °C, heart rate and oxygen saturation levels were stable while the patient was breathing room air (21 % oxygen). The blood pressure was 79/55 mmHg; the weight was 3180 g (23rd percentile), the length was 50 cm (32nd percentile), the head circumference 34 cm (22nd percentile).

On physical examination, the infant appeared well and comfortable. The heart rate was regular, heart sounds were normal. At rest, there were no signs of respiratory distress; the respiratory rate was 55 breaths per minute, and both lungs were equally ventilated without any noticeable rales or rhonchi. When agitated, the patient showed signs of respiratory distress with intercostal, suprasternal and supraclavicular retractions; inspiratory stridor was noticeable. The remainder of the examination was normal.

A nasogastric tube was inserted for enteral feedings and the patient was given nil by mouth until further diagnostic evaluation. Upon admission, a chest X-ray revealed very discrete bilateral opacification of both lungs (right > left) consistent with systemic infection. However, routine laboratory test results were repeatedly within normal range and did not indicate inflammation. Echocardiography showed no abnormal findings. A routine ultrasound of the brain revealed no pathologies; an amplitude-integrated electroencephalogram was appropriate for age without signs of seizure activity.

**Fig. 1:**
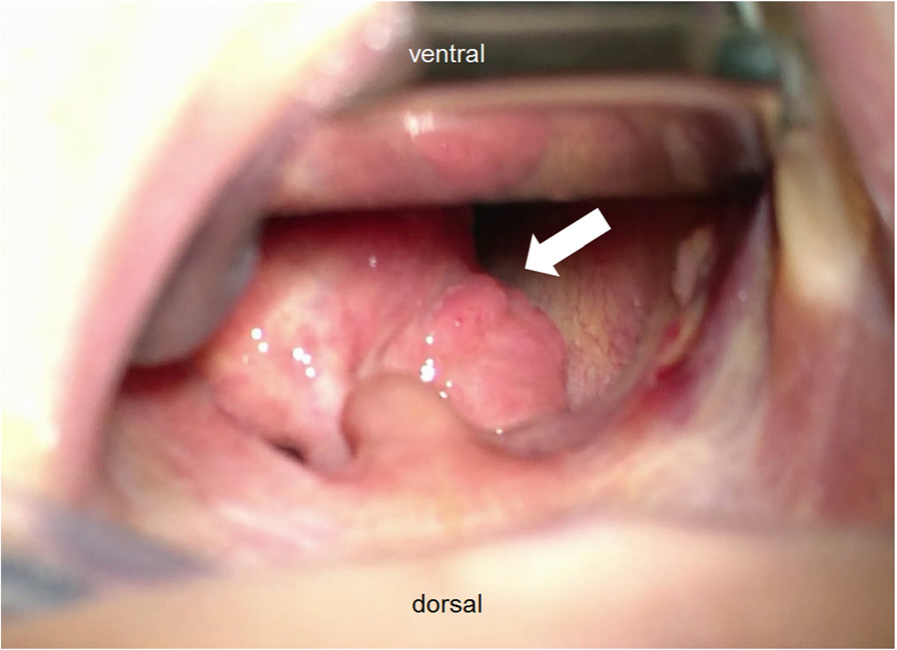
Intraoperative view of the pharynx prior to laser resection. When examining the surgical site, a protruding pediculated mass covered by mucous membranes (indicated by arrow) is clearly visible below the uvula. The endotracheal tube is still in position at that time, but cannot be readily seen due to a space-occupying effect of the tumor

**Fig. 2:**
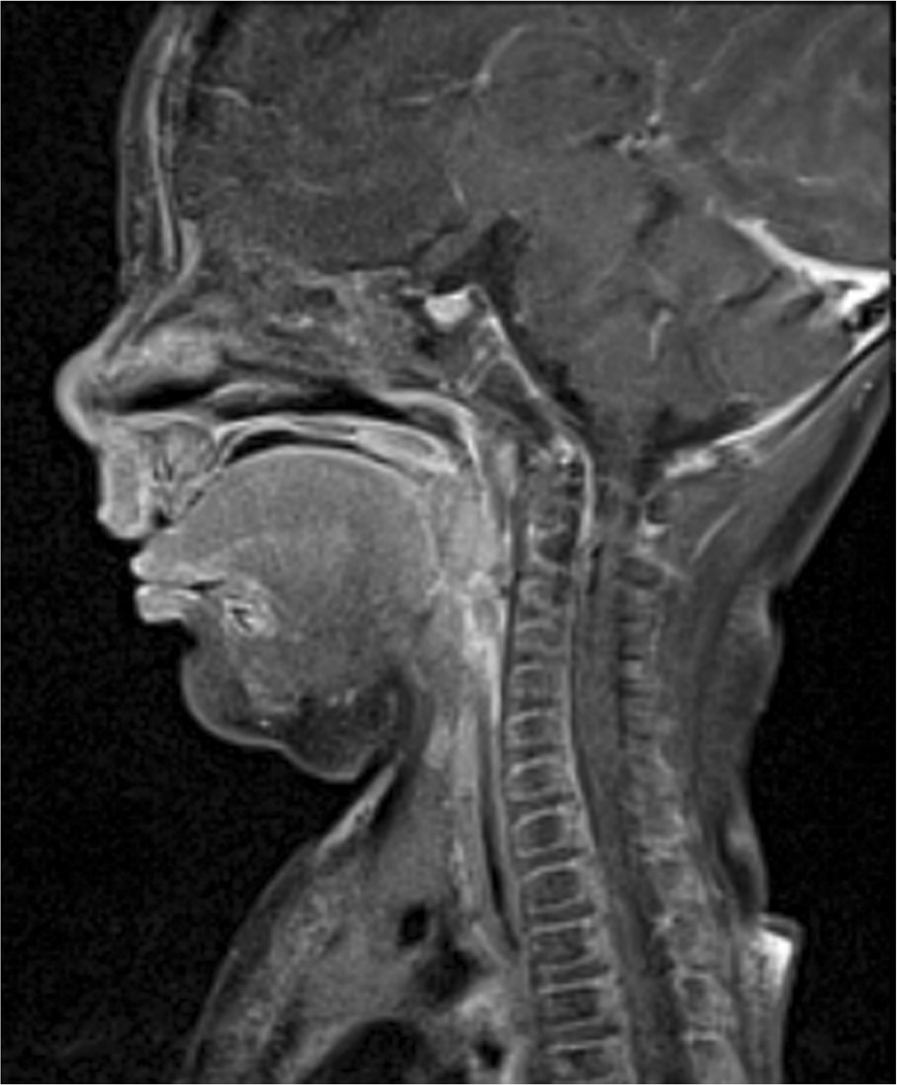
Magnetic resonance imaging of the head and neck region. Sagittal magnetic resonance image (T1-TSE) after contrast showing a pediculated 6.6 x 17.4 x 10 mm muscle-isointense lesion with a moderate uptake of contrast agent at the level of the second and third cervical vertebrae

**Fig. 3:**
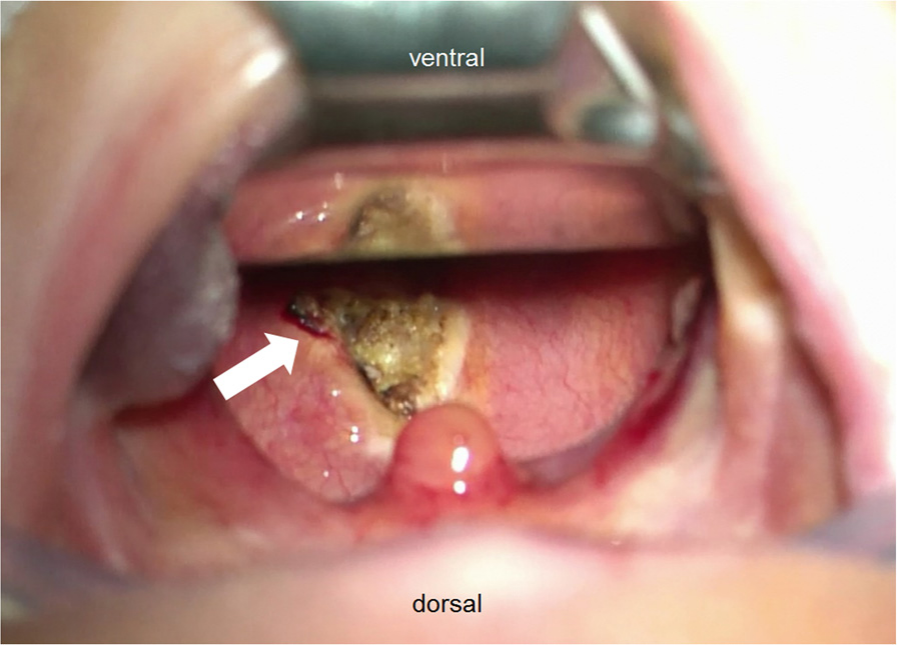
Intraoperative view of the pharynx after laser resection. The pediculated tumor has been successfully removed by laser resection. Minimal residual bleeding can be noted on the left ventrolateral resection margin (indicated by *arrow*)

**Fig. 4:**
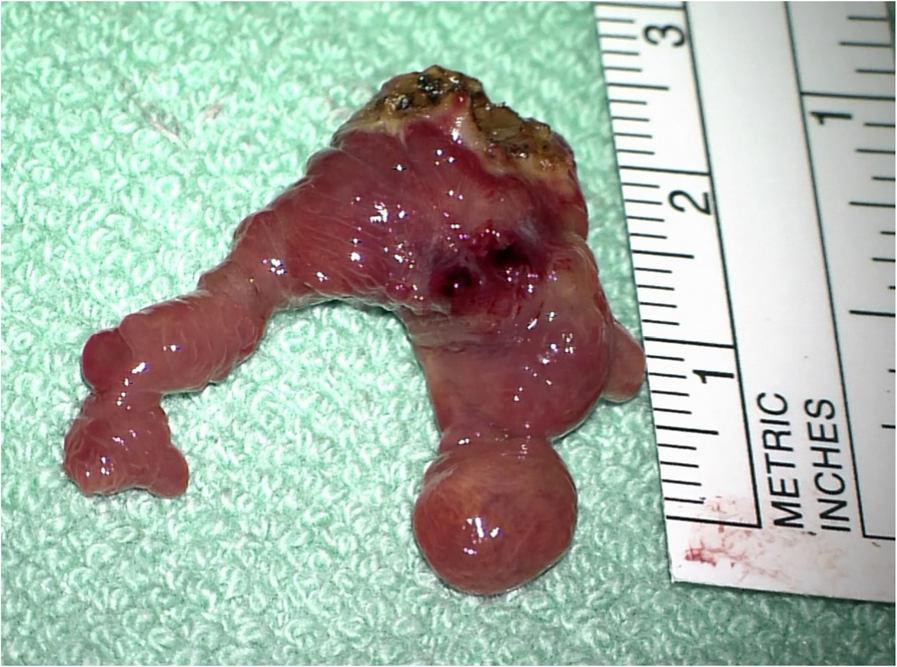
Surgical specimen. A double-branched tumor covered by mucous membranes measuring approximately 28 x 21 x 16 mm is removed by laser resection. Residual bleeding and thermic damage can be noted on the surface

**Fig. 5:**
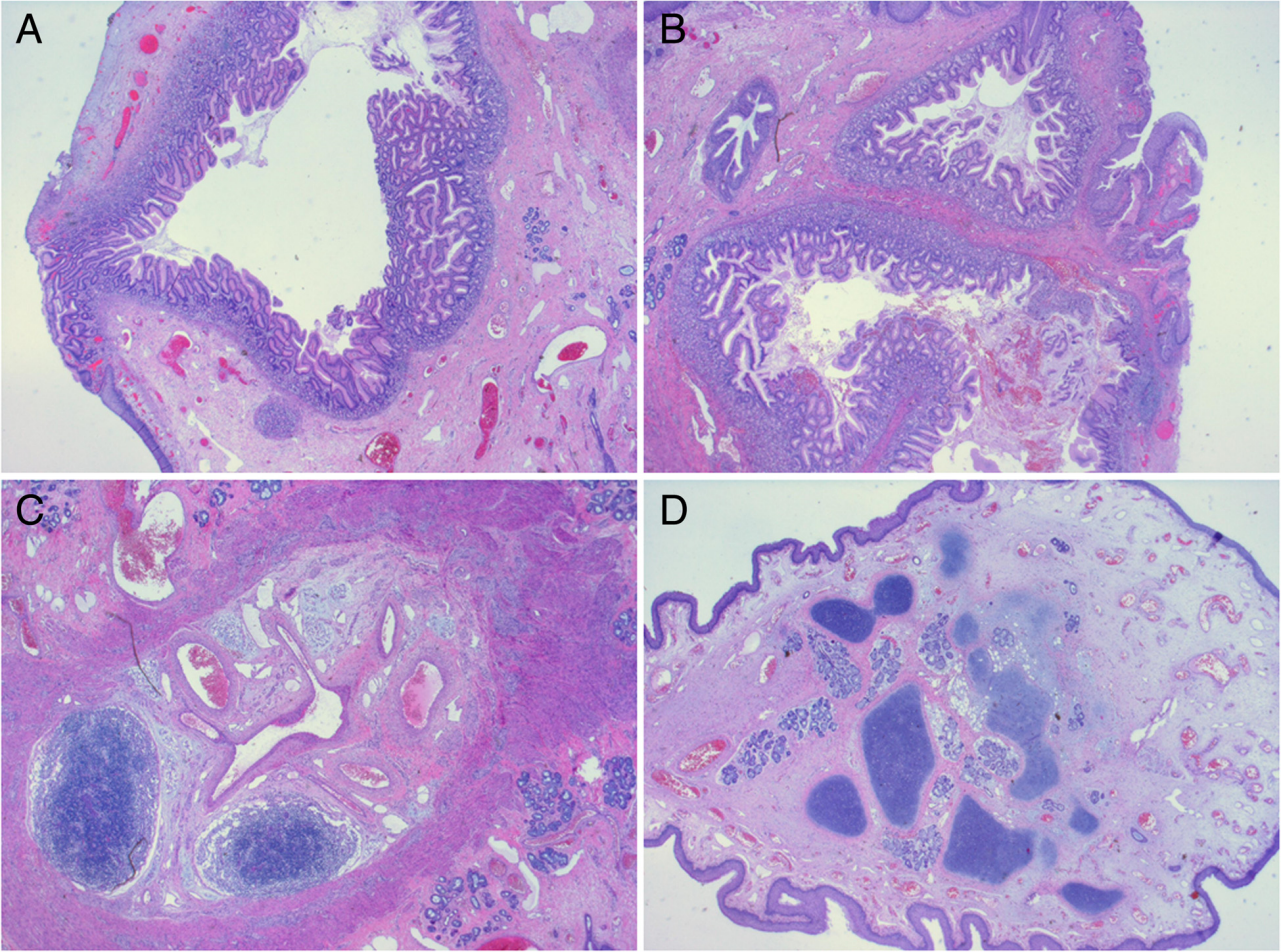
Histopathology. Histopathological examination identified several cystic structures lined with either squamous or columnar epithelium, gastric type, surrounded by fibroconnective and muscular stroma (**a**, **b**). In addition foci of lymphatic tissue (**c**) and small mucinous glands admixed with hyaline cartilage (**d**) were seen. Findings were consistent with a mature teratoma. Signs of malignancy were absent

On the sixth day of life, flexible endoscopy was performed and revealed a mucous protrusion of the dorsal oropharynx (Fig. 1). A mediastinal ultrasound and an X-ray swallow examination with a contrast agent were consistent with a space-occupying cystic lesion in the lower pharynx/upper esophagus. No associated anomalies could be detected by an abdominal ultrasound examination. On the seventh day of life, the patient was electively intubated and underwent magnetic resonance imaging, which showed a pediculated soft-tissue-isointense lesion of approximately 6.6 × 17.4 × 10 mm located at the dorsal oropharynx (Fig. 2). The following day, this lesion was removed transorally by potassium titanyl phosphate (KTP) laser resection and processed for histological evaluation (Fig. 3). Macropathology was consistent with a mature teratoma (Fig. 4). This diagnosis was subsequently confirmed by histopathology (Fig. 5). Signs of malignancy were absent.

The patient was extubated postoperatively without any complications, was subsequently hemodynamically stable and showed an uneventful further hospital course. Oral feedings could be re-initiated on the first postoperative day and were well tolerated. Parenteral nutrition was discontinued on the eleventh day of life. The patient was discharged after 15 days in hospital. Routine follow-up examinations after 3, 6 and 9 months showed no recurrence of the tumor, no abnormal physical findings and an age-appropriate development.

## Conclusions

A teratoma of the oropharynx is a highly unusual, but potentially life-threatening cause of inspiratory stridor and respiratory distress in the newborn. This case highlights the importance of an early and complete interdisciplinary workup of inspiratory stridor in the newborn, including non-invasive and invasive imaging techniques such as flexible endoscopy, in order to prevent complete airway obstruction and a potentially fatal outcome. In our case, early diagnosis and definitive surgical treatment were crucial.

## Consent

Written informed consent was obtained from the patient’s parents for publication of this case report and any accompanying images. A copy of the written consent is available for review by the editor of this journal.

## Abbreviation

KTP: potassium titanyl phosphate
